# Contribution and fate of maize residue-^15^N and urea-^15^N as affected by N fertilization regime

**DOI:** 10.1371/journal.pone.0210176

**Published:** 2019-01-08

**Authors:** Wencheng Ding, Shutian Li, Ping He, Shaomin Huang

**Affiliations:** 1 Ministry of Agriculture Key Laboratory of Plant Nutrition and Fertilizer, Institute of Agricultural Resources and Regional Planning, Chinese Academy of Agricultural Sciences (CAAS), Beijing, PR China; 2 International Plant Nutrition Institute (IPNI) China Program, CAAS-IPNI Joint Lab for Plant Nutrition Innovation Research, Beijing, PR China; 3 Institute of Plant Nutrition and Resource Environment, Henan Academy of Agricultural Sciences, Zhengzhou, PR China; Oak Ridge National Laboratory, UNITED STATES

## Abstract

Increasing amounts of crop residues are being returned to croplands. Understanding nitrogen (N) availability in crop residues under various N fertilization regimes is important in optimizing N management. Pot experiments were conducted to investigate the contribution, fate and residual effects of urea and maize residue-N using a ^15^N isotope technique. Four N regimes were tested: three basal–topdressing ratios of N applied as urea (100–0, 75–25, 50–50) and one basal application of 75% N as urea and 25% N as manure (75U+25M). An average of 31.4% wheat N uptake was derived from urea, 9.2% from maize residue, and 59.5% from the soil in the first season. During the growing stages of wheat, maize residue contributed 0.3–4.8% and 3.1–13.2% to soil mineral and microbial biomass N, respectively, and those originated from urea was 1.0–4.2% and 4.6–16.8%, respectively. Regarding the fate of urea and residue-N after the first season, 35.9% and 16.9% of urea-^15^N and residue-^15^N were recovered by wheat, 28.1% and 46.9% remained in the soil, and 36.0% and 36.2% were lost. The contribution of urea to crop N uptake and N recovery efficiency increased, while that of residue-N decreased with increasing proportion of topdressing N. Substituting 25% mineral N with manure decreased the urea-^15^N loss without negative effects on crop dry matter and N uptake. Residual urea-^15^N and maize residue-^15^N from the previous season contributed 3.9% and 3.0% to summer maize N uptake. Additionally, 29.3% of residue-^15^N remained in the soil after the second season, while only 18.6% of urea-^15^N remained. Our study suggests that fertilizer and crop residue are actively involved in soil N transformation and plant N nutrition, emphasizing the capacities of organic residues to sustainably supply nutrients. Considering the utilization of both N fertilizer and maize residue, we may suggest a 75–25 split in N fertilizer application, but more appropriate options need to be further assessed under different cropping systems.

## Introduction

Most agricultural soils are depleted of indigenous N and thus require large quantities of N to be applied in the form of mineral fertilizer. Annually, China consumes more than one-quarter of the total mineral N fertilizer used worldwide [1]. Decreasing fertilizer N loss and increasing N use efficiency are essential not only for financial consideration but also for environmental protection. It has been extensively reported that N fertilizer is largely inefficient [2–4], and the consequent high N loss via the runoff, leaching and greenhouse gas emissions has serious implications for surface/ground water contamination and climate change [5–8]. Optimizing fertilization management has been showed as an effective means to improve agricultural productivity and mitigate the negative effects of fertilizer N losses [9].

One focus area of the Chinese government’s recent policy of “Zero Growth of Chemical Fertilizer Use by 2020” (http://jiuban.moa.gov.cn/zwllm/tzgg/tz/201503/t201503184444765.htm) is understanding how organic residues can be better used to offset partial mineral fertilizer. Utilization of organic N sources, such as crop residues and farmyard manure, is considered an effective means to cycle nutrients and buildup soil organic matter. Returning crop residues to fields is becoming a routine practice worldwide as an efficient type of conservation agriculture [10]. China produces approximately 925 million tons of crop residues annually [11], with 34.2%, 33.1% and 14.5% of the total nutrient resources originating from maize, rice, and wheat residues, respectively [12]. Maize residue generally contains approximately 80 kg N ha^–1^, which may act as an important N source for soil N pools and subsequent crop N uptake [13–14]. Winter wheat-summer maize rotation is the dominant crop system in the North China Plain, where Fluvo-aquic soils have poor soil fertility because of its calcareous nature, frequent tillage and lack of organic matter [8]. Crop residue retention can effectively improve crop productivity, soil fertility, and enhance nutrients use efficiency by recycling them through the soil [15–16]. A meta-analysis showed that crop residue retention increased yield by 5.2%, 6.4% and 5.6%, respectively for rice, wheat and maize in China [17]. Nevertheless, increasing crop yield with crop residue retention depends on many other practices, such as water and fertilization management. In addition, numerous factors, such as inherent N content, C/N ratio, lignin and polyphenol concentrations in crop residues, soil properties and climatic conditions, play important roles in decomposition and nutrient release from crop residue [18–20].

N mineralization and immobilization are two important processes closely related to N bioavailability of fertilizers and crop residue applied to the soil [8,21], which can be partially described by the dynamics of soil mineral N and microbial biomass N. Following the addition of crop residue into soils, a rapid decrease in soil nitrate content and increases in soil microbial biomass C and N were observed in the early stages of an incubation experiment [22–23]. Immobilization of fertilizer and crop residue N in soil, especially that immobilized into a stable fraction of soil organic matter components, may contribute to the sustainability of soil fertility in the long term [24–25]. Ding et al. [26] reported that more maize residue-N was transformed into amino sugars in response to lower inorganic N addition in the early stages of crop residue degradation, while the trend was reversed in the later stages. These findings suggest that a higher availability of inorganic N could delay the process of transformation of plant-N into microbial residues during the mineralization of plant residues. Moreover, soil microbial communities play a significant role in nutrient cycling and decomposition of residues [27]. They are valuable indicators of soil quality and ecosystem functioning [15], which can be strongly influenced by the practices of fertilizer and crop residue management [28].

Most studies calculate N recovery efficiency using the N-difference method (i.e. the differences between N-fertilized and control plots). However, this approach may cause errors (usually overestimating N recovery efficiency) because considerable differences exist between non-fertilized and fertilized treatments in terms of crop N uptake and soil N pool [8,29]. The ^15^N isotope dilution technique is a promising means of tracing the distribution of labeled-^15^N in plant-soil N cycling, providing precise information on crop N uptake from a specific source [30–32]. Large differences exist in total ^15^N recovery of plants or soils between N application in the forms of fertilizer and crop residues. The crop N recovery from organic N sources is usually lower than that from inorganic N sources under short-term conditions [33], because of the inferior availability of organic N relative to mineral N. Results from Chen et al. [34] showed that 17.2% of the fertilizer-^15^N and 12.0% of rice residues-^15^N were recovered by wheat with 33.46% and 85.64% of fertilizer-^15^N and residue-^15^N, respectively, left in the soil after the first growing season. Although fertilizer N appeared to be more readily available to crops, crop residue can replenish the soil N pool, especially soil organic N, with a much greater degree than fertilizer N. Therefore, crop residues are better sources for sustaining soil fertility [35].

Understanding the dynamics of mineral fertilizer- and organic-N in soil-plant systems under different N management is of considerable importance in developing optimal fertilization practices for minimizing N loss while maximizing use efficiency [9,36]. Thus, using ^15^N-labeled maize residue and urea, pot experiments were carried out to quantify the contribution and determine the fate of crop residue and fertilizer in crop N nutrition and soil N pools in both the current and following seasons, as well as to identify the optimal basal-topdressing ratio of N fertilization under crop residue retention. Considering the importance of N addition on residue decomposition and split-application of N fertilizer is widely approved, we hypothesize that different N regimes will have a strong influence on the availability of fertilizer-N and residue-N. Also, substituting mineral N with organic N is expected to mitigate N loss by enhancing the immobilization of mineral N without negatively affecting yield.

## Materials and methods

### Experimental design and materials

Parallel experiments were conducted with a combination of ^15^N-labeled urea and non-labeled maize residue, and a combination of ^15^N-labeled maize residue and non-labeled urea, respectively. Four N regimes (three basal-topdressing ratios and one organic substitution) were tested in the two parallel experiments: basal application of 100% N as urea (100–0), basal application of 75% N and topdressing application of 25% N as urea (75–25), basal application of 50% N and topdressing application of 50% N as urea (50–50), and basal application of 75% N as urea and of 25% N as composted pig manure (N-P_2_O_5_-K_2_O: 1.86%-3.11%-0.85%) (75U+25M). A treatment without N served as a control (CK). Basal N was applied to the pots before sowing, and topdressing N was applied at the beginning of stem elongation. A total of nine treatments were arranged based on a randomized complete block design with nine replicating pots. All treatments were applied with non-labeled urea and non-labeled wheat straw in the subsequent summer-maize season.

The atom fraction ^15^N (%Atom ^15^N) of ^15^N-labeled urea was 10.2%. The residues used in the pot experiments included ^15^N-labeled maize residue, non-labeled maize residue and non-labeled wheat straw at a rate of 20g pot^–1^, ground and sieved through 0.5 mm. The basic properties of these residues are given in Table 1. A single rate of total fertilizer application (i.e., 100 mg N, 50 mg P_2_O_5_ and 40 mg K_2_O per kg soil) was applied for all treatments except CK. Urea, potassium chloride, and monocalcium phosphate were applied as mineral N, P, and K fertilizer sources, respectively. The fluvo-aquic soil used in the experiment was obtained from a field (0–20 cm layer) dominated by a winter wheat-summer maize rotation system in Henan province. The soil contained 9.2 g kg^–1^ organic matter, 0.82 g kg^–1^ total N, 6.7 mg kg^–1^ NH_4_^+^-N, 15.8 mg kg^–1^ NO_3_^–^-N, 22.9 mg kg^–1^ available P and 112.2 mg kg^–1^ available K, and had a pH of 8.0 (1:2.5, soil:H_2_O ratio). The soil was air dried, sieved through 2 mm, and adequately homogenized before loading into the pots.

**Table 1:** The properties of crop residues applied in experiments.

Materials	Total C (%)	Total N (g·kg^–1^)	Total P (g·kg^–1^)	Total K (g·kg^–1^)	%Atom ^15^N (%)	C/N
maize residue ^a^	41.18	13.59	1.89	27.11	43.21	30.03
maize residue ^b^	41.56	13.34	1.82	27.35	0.37	31.15
wheat straw ^b^	40.53	8.08	0.88	31.52	0.37	50.16

^a 15^N-labeled

^b^ non-labeled

### Pot experiment and sampling

The pot experiments were conducted in a net house with a glass roof at Chinese Academy of Agricultural Sciences (N39°57′, E116°19′) from October 2014 to October 2015, during which time winter wheat (*Triticum aestivum* L.) and summer maize (*Zea mays* L.) were planted successively. Five kilograms of the soil were loaded into a plastic and non-leaching pot with 20 cm in diameter and 25 cm in height. Manure and maize residue were adequately homogenized with soil before sowing (October 9, 2014). Mineral fertilizers were dissolved using deionized water and poured into the soil. Each pot was sown with 20 winter-wheat seeds and 15 strains were kept when the seedlings emerged. Following the harvest of wheat, five summer-maize seeds were sown as a succeeding crop on June 6, 2015, and three strains were kept after the emergence of seedlings. During the experiment, soil moisture content was adjusted to approximately 60% of the water holding capacity by regularly adding deionized water.

The soil and plant samples of each treatment were collected at after-dormancy (May 9, 2015), heading (April 23, 2015), maturity stages (June 1, 2015) of winter wheat, and maturity stage of summer maize (October 5, 2015) respectively. Three pots were destructively sampled for each treatment: all soils in each sampling pot were poured out and homogenized completely, after which 100 g subsamples were collected to determine the soil total N, mineral N and microbial biomass N content, and their respective atom% ^15^N. Plants were divided into grain, straw and roots if available, and each part was oven dried at 65°C to a stable weight, ground and then sieved through 0.25 mm.

### Chemical analysis

Soil organic matter was determined using the K_2_Cr_2_O_7_ titration method. Soil available P was extracted with 0.5 M NaHCO_3_ (pH 8.5) and then determined using the Olsen method [37], and soil available K was extracted with 1 M ammonium acetate (pH 7.0), and measured by atomic absorption spectrometry (NovAA300, Analytik Jena AG). The total N content and the atom% ^15^N of soils and plants were determined using a stable isotope ratio mass spectrometer (Isoprime100 and Vario Pyrd/Cube, Elementar, Germany). Soil mineral N content (N_min_: NH_4_^+^-N and NO_3_^–^-N) was extracted with 1M KCl and determined using a flow injection analyzer (FLA star 5000 Analyzer, Foss, Denmark). Soil microbial biomass N content (MBN) was determined by chloroform fumigation-extraction protocol, extracted with 0.5M K_2_SO_4_, and analyzed using a multi N/C analyzer (Multi N/C 3100/HT1300, Analytik Jena AG, Germany). The atom% ^15^N of N_min_ and MBN was determined using a stable isotope ratio mass spectrometer (Isoprime100 and Vario Pyrd/Cube, Elementar, Germany).

### Enzyme activity and PLFA analysis

The soil enzymes involved in C-transformation (β-cellobiosidase, β-glucosidase, β-xylosidase, and N-acetyl-β-glucosaminidase), N-transformation (urease, aminopeptidase, and N-acetyl-β-glucosaminidase), and oxidoreductases (peroxidase and phenol oxidase) were analyzed. All enzyme activities (except urease, phenol oxidase, and catalase) were quantified according to fluorescence-based protocols, and phenol oxidase and peroxidase were measured colorimetrically in a clear 96-well microplate [38–39]. Urease activity was determined according to Kandeler and Gerber [40].

Soil microbial community composition and microbial biomass were determined by phospholipid fatty acid (PLFA) analysis as described in detail by Wang et al. [39]. Bacteria (Gram-positive and Gram-negative), fungi and actinomycete groups were distinguished from total PLFA according to previously published PLFA biomarker data [39,41], and the abundance of each group was indicated by the ratios of their concentrations to total PLFA concentration.

### Calculations

The percentage of crop N, soil total N or N_min_ derived from ^15^N-labeled residue (%Ndfr) and from ^15^N-labeled urea (%Ndfu) was calculated as follows:

$$\%Ndfr=100\times \frac{a‑c}{b_{r}-c}$$

$$\%Ndfu=100\times \frac{a‑c}{b_{u}-c}$$

where, *a* is the atom% ^15^N of crop N, soil N or N_min_, *b*_*r*_ and *b*_*u*_ represent the atom% ^15^N of ^15^N-labeled residue and ^15^N-labeled urea, respectively, and *c* is the atom% ^15^N of naturally occurring N.

The percentage of MBN derived from ^15^N-labeled residue (%Ndfr) or^15^N-labeled urea (%Ndfu) was calculated as follows:

$$\%Ndfr,u=100\times \frac{d(d^{'}‑c)-e(e^{'}-c)}{\left(d-e)(b_{r,u}-c\right)}$$

where *d* and *e* represent fumigated and no fumigated MBN concentration, respectively, *d’* and *e’* are the atom% ^15^N of fumigated and non-fumigated MBN, respectively. *b*_*r*,*u*_ and *c* are the same as above.

N recovery efficiency of ^15^N-labeled residue (REN_r_) or ^15^N-labeled urea (REN_u_) by crop:

$$REN_{r,u}=100\times \frac{f\times \%Ndfr,u}{g_{r,u}}$$

where *f* is the crop uptake N content, %Ndfr,u represents the percentage of crop N derived from ^15^N-labeled residue or ^15^N-labeled urea, and *g*_*r*,*u*_ is N content of ^15^N-labeled residue or urea, respectively.

N residual rate of ^15^N-labeled residue (RRN_r_) or ^15^N-labeled urea (RRN_u_) in soil:

$$RRN_{r,u}=100\times \frac{h\times \%Ndfr,u}{g_{r,u}}$$

where *h* is the soil total N content, %Ndfr,u represents the percentage of soil total N derived from ^15^N-labeled residue or ^15^N-labeled urea, and *g*_*r*,*u*_ is the same as above.

N loss rate of ^15^N-labeled residue (LRN_r_) or ^15^N-labeled urea (LRN_u_):

$$LRN_{r,u}=100-REN_{r,u}-RRN_{r,u}$$

### Statistical analysis

Differences between treatment groups with N regime as an independent factor were tested using SPSS version 21. The data were firstly tested for equal variance, and then means were compared using LSD (Least Significant Difference; at 0.05 level of probability) test by one-way ANOVA analysis.

## Results

### Dry matter production and N uptake of two successive crops

Compared to CK, N fertilization significantly increased total dry matter and N uptake in most instances, but no significant difference was observed among N regimes throughout the winter wheat growth stages (Table 2). For subsequent summer maize, N application still significantly increased total dry matter compared to CK, and differences appeared among different N regimes. The highest total dry matter and N uptake by maize were obtained in 50–50 treatment, closely followed by 75–25 treatment. Relative to spilt application of N, treatments with all N applied as basal (75U+25M and 100–0) showed negative effects on both dry matter and N uptake.

**Table 2:** Total dry matter (root, shoot, and grain) and N uptake of winter wheat and summer maize.

Treatments	Winter wheat						Summer maize	
	After-dormancy		Heading		Maturity			
	Dry matter (g pot^–1^)	N uptake (mg pot^–1^)	Dry matter (g pot^–1^)	N uptake (mg pot^–1^)	Dry matter (g pot^–1^)	N uptake (mg pot^–1^)	Dry matter (g pot^–1^)	N uptake (mg pot^–1^)
CK	2.8b	107.7a	16.6b	264.9b	23.6b	301.2b	14.3c	106.1d
100–0	3.9a	136.7a	19.1a	465.7a	27.4a	518.8a	41.3b	293.8c
75–25	3.2ab	120.2a	19.5a	462.2a	27.1a	486.7a	56.3a	422.1b
50–50	3.2ab	117.7a	19.6a	465.8a	27.6a	543.1a	56.8a	458.2a
75U+25M	3.6a	143.0a	19.5a	428.8a	28.3a	482.1a	45.5b	302.3c

CK: no N applied; 100–0: 100% basal N by urea; 75–25: 75% basal N and 25% topdressing N by urea; 50–50: 50% basal N and 50% topdressing N by urea; 75U+25M: 75% basal N by urea and 25% basal N by manure. Numbers followed by different letters in the same column showed significant differences at *p* < 0.05.

### Effect of N regime on the percentages of crop N derived from ^15^N-labeled residue and ^15^N-labeled urea

The percentages of N uptake by the whole wheat plants (grain + straw + root) derived from ^15^N-labeled residue (%Ndfr), ^15^N-labeled urea (%Ndfu) and other sources (%Ndfs) (mainly referred to the soil for treatments applied urea only; soil and manure for 75U+25M treatment) ranged 8.3–9.9%, 23.8–35.9% and 55.9–66.5%, respectively (Table 3). Variations were observed on N uptake of different plant organs from different N sources, i.e., grain and roots were found with higher %Ndfu and %Ndfr while straw with higher %Ndfs. For N uptake dynamics, N regime had no significant influence on residue’s contribution to N uptake in the after-dormancy stage of wheat, while after that, there was a significant decrease of %Ndfr with increasing proportion of topdressing N application and the %Ndfs of 75U+25M was significantly higher than those of 75–25 and 50–50 treatments at the heading and maturity stages. Contrary to %Ndfr, %Ndfu decreased in the after-dormancy stage but increased in the heading and maturity stages with an increasing proportion of topdressing N. The 75U+25M treatment reduced the contribution of ^15^N-labeled urea to wheat N uptake during the late growth stages. Because of differences in the contribution of ^15^N-labeled residue and ^15^N-labeled urea, the wheat N uptake from other sources showed the following tendency: 75U+25M > 100–0 > 75–25 > 50–50.

**Table 3:** The percentages of plant N derived from ^15^N-labeled residue (%Ndfr), ^15^N-labeled urea (%Ndfu), and other sources (%Ndfs).

	Winter wheat										Summer maize		
Treatments	After-dormancy			Heading			Maturity						
	Straw	Root	Plant	Straw	Root	Plant	Grain	Straw	Root	Plant	Shoot	Root	Plant
%Ndfr (%)													
100–0	9.7a	9.0b	9.6a	10.0a	9.2a	9.9a	10.5a	8.2a	10.0a	9.9a	2.6a	4.6ab	3.0ab
75–25	10.4a	10.1a	10.4a	9.1b	8.7b	9.1b	9.7b	7.9a	9.2b	8.9b	2.4a	3.9b	2.7b
50–50	10.8a	10.0a	10.6a	8.3c	8.3c	8.3c	8.7c	7.5a	8.6c	8.3c	2.6a	4.3b	2.9b
75U+25M	9.8a	9.1b	9.70a	10.3a	9.3a	10.2a	10.5a	7.6a	10.2a	9.7a	2.9a	5.1a	3.4a
%Ndfu (%)													
100–0	35.5a	31.3a	35.0a	34.4c	30.1a	33.9c	33.9c	28.6a	29.7b	31.9c	3.4a	6.4a	4.0ab
75–25	28.8b	27.3b	28.3b	37.8b	31.7a	37.2b	36.0b	30.0a	30.7a	34.2b	3.6a	6.1ab	4.1a
50–50	17.8c	21.2d	18.6d	40.3a	33.0a	39.6a	38.7a	29.5a	31.0a	35.9a	3.2a	5.7b	3.6b
75U+25M	26.1b	23.4c	25.6c	27.0d	23.9b	26.7d	25.3d	20.0b	22.4c	23.8d	3.2a	5.8b	3.8ab
%Ndfs (%)													
100–0	54.8c	59.7c	55.5c	55.6b	60.7b	56.2b	55.6b	63.2b	60.3b	58.1b	94.0a	89.0b	93.0b
75–25	60.8b	62.6b	61.4b	53.1c	59.6b	53.7c	54.2c	62.1b	60.1b	56.9c	93.9a	89.9a	93.1ab
50–50	71.4a	68.8a	70.8a	51.4c	58.7b	52.2c	52.6d	63.0b	60.4b	55.9d	94.3a	90.0a	93.5a
75U+25M	64.1b	67.5a	64.7a	62.7a	66.8a	63.2a	64.2a	72.3a	67.4a	66.5a	93.9a	89.1b	92.8b

100–0: 100% basal N by urea; 75–25: 75% basal N and 25% topdressing N by urea; 50–50: 50% basal N and 50% topdressing N by urea; 75U+25M: 75% basal N by urea and 25% basal N by manure. Numbers followed by different letters in the same column showed significant differences at *p* < 0.05.

During the summer-maize season, the contribution of residual ^15^N-labeled residue and ^15^N-labeled urea to N uptake was significantly reduced relative to the first season, when %Ndfr and %Ndfu were 2.7–3.4%, and 3.6–4.1%, respectively, for the whole plants (Table 3). The main source of N uptake was derived from the soil and non-labeled N sources applied during the current season. The 75U+25M treatment showed the highest %Ndfr when compared to treatments receiving only urea. The %Ndfu of 75–25 treatment was significantly higher than that of 50–50 treatment.

### Effect of N regime on N recovery, residual and loss of ^15^N-labeled residue and ^15^N-labeled urea

**Fig 1:**
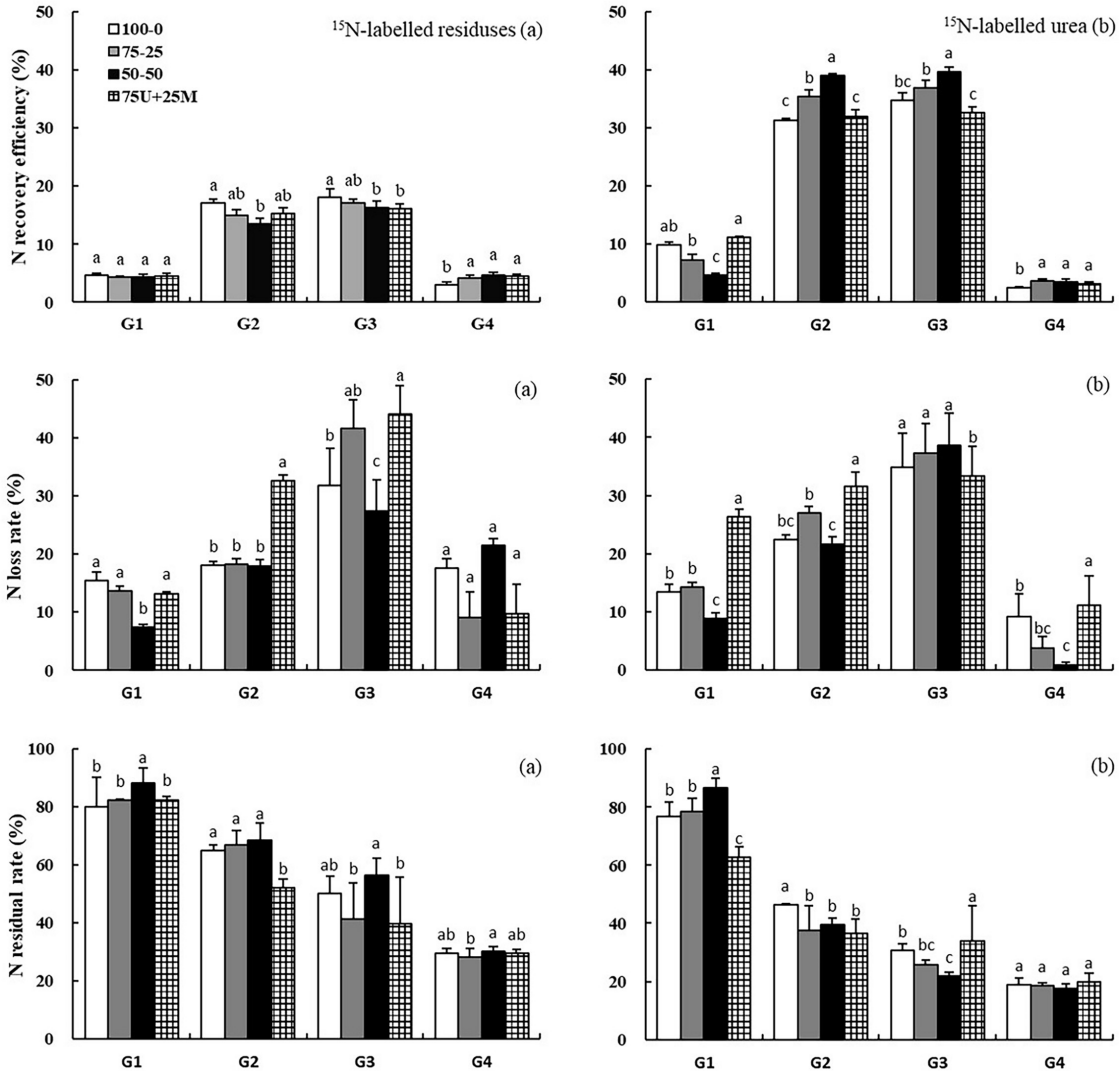
**Effect of N regime on nitrogen recovery efficiency, residual rate and loss rate for** ^**15**^**N-labelled residue (a) and** ^**15**^**N-labelled urea (b), respectively.** G1, G2, G3, and G4 represent the after-dormancy, heading and maturity stages of winter wheat, and maturity stage of summer maize, respectively. Vertical bars represent the standard errors and different letters above the bars indicate significant differences between treatments at *p* < 0.05.

On average, N recovery efficiency (REN_r_) and loss rate (LRN_r_) of ^15^N-labeled residue increased from 4.5% to 16.9% and 12.4% to 36.2%, and consequently, the residual rate of ^15^N-labeled residue (RRN_r_) decreased from 83.1% to 47.0% from after-dormancy to maturity of wheat (Fig 1A). In the second season, the averaged REN_r_, LRN_r_ and RRN_r_ were 4.1%, 14.5% and 29.3%, respectively. The REN_r_ decreased with increasing proportion of topdressing N in the first season, while a completely opposite result was observed in the second season. The REN_r_ of 75U+25M treatment (16.2%) was significantly lower than that of 100–0 treatment (18.1%), but an opposite result was observed in the second season. The highest LRN_r_ was observed in 75U+25M treatment in the first season, when 44.1% of residue-N was lost.

N recovery efficiency (REN_u_) of ^15^N-labeled urea increased sharply from 8.2% after dormancy stage to 34.4% at the heading stage, after which slight increases occurred until wheat maturity (35.9%) (Fig 1B). On average, the loss (LRN_u_) and residual rate (RRN_u_) of ^15^N-labeled urea were 36.0% and 28.1% after the first season. During the second season, only 3.1% for REN_u_ was observed, while LRN_u_ and RRN_u_ were 6.3% and 18.6%, respectively. Contrary to REN_r_, when more urea was applied as topdressing, a significant increase of REN_u_ was observed in the heading and maturity stages of wheat. The REN_u_ and LRN_u_ of 75U+25M treatment (32.6% and 33.4%) was significantly lower than those of 75–25 (36.9% and 37.3%) and 50–50 (39.6% and 38.6%) treatments. A lower REN_u_ was observed for 100–0 treatment compared to the other three treatments in the second season.

### Effect of N regime on the contribution of ^15^N-labeled residue and ^15^N-labeled urea to soil mineral and microbial biomass N

The percentages of soil total N, mineral N (N_min_) and microbial biomass N (MBN) derived from ^15^N-labeled residue (%Ndfr) were 3.3–5.3%, 0.3–4.8% and 3.1–13.2%, respectively; those from ^15^N-labeled urea (%Ndfu) were 3.7–9.0%, 1.0–4.2% and 4.6–16.8%, respectively, throughout the wheat growing stages (Table 4). No significant difference was observed on %Ndfr of soil total N between different N regimes. The %Ndfr of N_min_ was significantly higher for 75U+25M treatment in the after-dormancy and heading stages compared to treatments without manure applied, while for the %Ndfr of MBN, 75U+25M treatment was significantly higher than that of 50–50 treatment only in the after-dormancy stage. The %Ndfu of total N showed a significant decrease with increasing proportions of topdressing N in the after-dormancy and maturity stages. The %Ndfu of N_min_ for 75–25 treatment was significantly higher than that of 75U+25M treatment, while the %Ndfu of MBN for 50–50 treatment was significantly higher than that of 75U+25M treatment.

**Table 4:** The percentages of soil total N, N_min_, and MBN derived from ^15^N-labeled residue (%Ndfr) and ^15^N-labeled urea (%Ndfu) during winter-wheat season.

Treatments	After-dormancy			Heading			Maturity		
	Total N	N_min_	MBN	Total N	N_min_	MBN	Total N	N_min_	MBN
%Ndfr (%)									
100–0	5.1a	1.0b	12.2ab	4.4a	0.3b	3.1a	4.3a	0.6a	3.7a
75–25	5.3a	0.7b	9.2ab	3.3a	0.6b	3.3a	3.5a	0.7a	4.1a
50–50	3.9a	0.8b	6.6b	4.6a	0.4b	3.7a	3.4a	0.8a	3.9a
75U+25M	5.2a	4.8a	13.2a	4.7a	2.0a	3.2a	3.3a	0.7a	4.3a
%Ndfu (%)									
100–0	9.0a	4.2a	32.9a	6.2a	2.3a	17.0a	5.6a	1.6ab	5.0ab
75–25	6.2b	2.3ab	16.8ab	5.1a	1.6a	12.3a	4.5b	1.8a	6.3ab
50–50	4.3c	1.5b	8.1b	5.3a	2.2a	9.9a	3.7c	1.5ab	7.0a
75U+25M	5.6bc	1.9b	15.0ab	5.2a	1.4a	5.7a	5.0ab	1.0b	4.6b

100–0: 100% basal N by urea; 75–25: 75% basal N and 25% topdressing N by urea; 50–50: 50% basal N and 50% topdressing N by urea; 75U+25M: 75% basal N by urea and 25% basal N by manure. Numbers followed by different letters in the same column showed significant differences at *p* < 0.05.

### Effect of N regime on soil enzyme activity and microbial community composition

The activities of eight soil enzymes involved in the transformation of soil C and N, and oxidoreductases were determined after wheat harvest (Table 5). The activities of enzymes involved in C transformation (β-glucosidase, β-cellobiosidase, β-xylosidase, and N-acetyl-β-glucosaminidase) showed similar trends among treatments (i.e., 100–0 > 50–50 > 75–25 > 75U+25M > CK). Urease activity in 75U+25M treatment and aminopeptidase activity in 75–25 treatment were significantly higher than those in other treatments except 50–50. For oxidoreductases, the activities of phenol oxidase and catalase decreased significantly compared to CK, while no differences were observed between the treatments with N application.

**Table 5:** The effect of N regime on soil enzyme activities (nmol g^–1^ h^–1^) after wheat harvest.

Treatments	β-glucosidase	β-cellobiosidase	β-xylosidase	N-acetyl-β-glucosaminidase	Urease	Amino- peptidase	Phenol oxidase	Catalase
CK	99.7c	20.7c	26.2b	7.1c	8.9c	389.6cd	4.1a	4.4a
100–0	158.4a	41.1a	49.8a	16.1a	11.9b	415.9bc	2.3b	1.8b
75–25	144.3ab	34.9b	47.5a	13.6b	12.3b	454.0a	2.5b	2.0b
50–50	148.2ab	38.0ab	47.1a	15.0ab	12.5ab	445.5ab	2.4b	1.8b
75U+25M	134.0b	34.0b	44.5a	13.8b	13.5a	367.1d	2.5b	2.2b

100–0: 100% basal N by urea; 75–25: 75% basal N and 25% topdressing N by urea; 50–50: 50% basal N and 50% topdressing N by urea; 75U+25M: 75% basal N by urea and 25% basal N by manure. Numbers followed by different letters in the same column showed significant differences at *p* < 0.05

**Fig 2:**
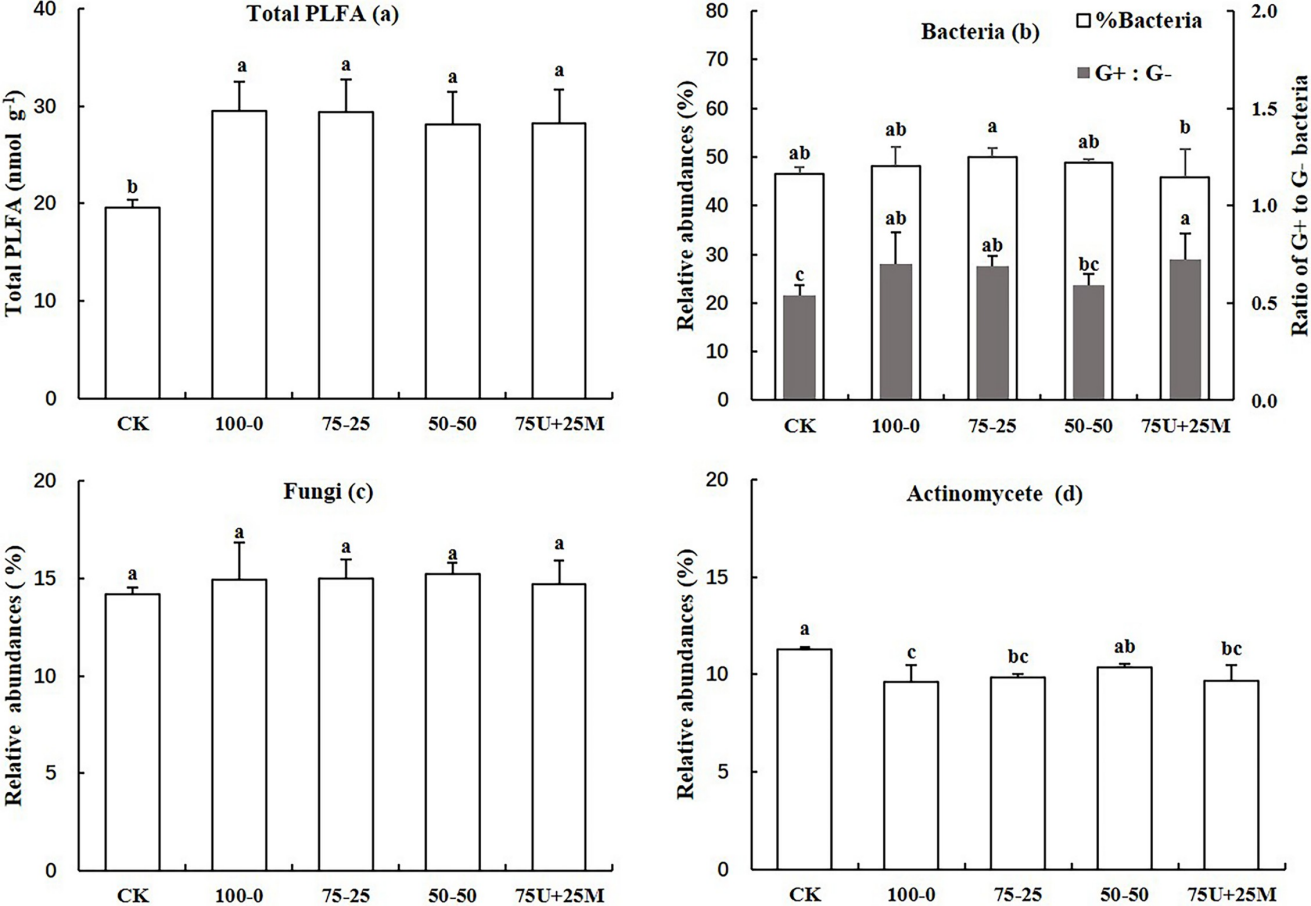
**Effect of N regime on total PLFA (a), the relative abundance of bacteria with the ratio of Gram positive to Gram-negative bacteria (b), fungi (c) and actinomycete (d).** CK: no N applied; 100–0: 100% basal N by urea; 75–25: 75% basal N and 25% topdressing N by urea; 50–50: 50% basal N and 50% topdressing N by urea; 75U+25M: 75% basal N by urea and 25% basal N by manure. Vertical bars represent the standard errors and different letters above the bars indicate significant differences between treatments at *p* < 0.05.

Total microbial biomass was quantified based on the total concentration of PLFA. N fertilization significantly increased total PLFA relative to CK, while no difference was observed between N regimes (Fig 2A). The relative abundance of 75–25 treatment was higher than that of 75U+25M treatment for bacteria, and the ratio of Gram-positive to Gram-negative bacteria increased for 75U+25M treatment compared to 50–50 and CK (Fig 2B). No significant differences were observed between treatments for fungi (Fig 2C). A decrease in the level of actinomycetes was observed in the N addition treatments compared to CK, while the relative abundance of 50–50 treatment was significantly higher than that of 100–0 treatment (Fig 2D).

## Discussion

### Contribution of maize residue and urea to crop N uptake and soil N pools

In addition to N mineralization from indigenous soil, fertilizer application and organic resource input are two principal N sources for maintaining crop productivity. The percentage of crop N derived from various N sources is an important index that provides some information regarding the mechanism of N uptake and the efficient utilization of a specific N source. We found the proportion of crop N derived from maize residue, urea and the soil were approximately 1:3:6 with little changes observed for different growth stages (Table 3), indicating that the soil and fertilizer are dominant factors of N contribution. As maize residue-N input (0.27 g per pot) was less than fertilizer-N (0.5 g per pot), the finding still confirmed that the residue is an important N source for agricultural systems. A similar result was observed in a pot experiment in which 7–10% of wheat N was found to originate from maize residues [42]. Another study conducted in a rice-wheat cropping system indicated that 17.2% and 12.0% of crop N were derived from N fertilizer and residues, respectively, during the first growing season [34]. These previous findings were similar to our investigation with respect to crop residue, while the results for N fertilizer were much lower than ours, which may have occurred because our study was conducted under pot conditions instead of field conditions. When investigating N fertilization regime, split-application of N fertilizer resulted in a higher contribution than totally basal application, with a greater ratio of topdressing N being associated with a higher contribution of fertilizer-N to crop N. Conversely, for maize residue, basal N increased the bioavailability of residue-N, implying that more mineral N application at an early stage will alleviate the immobilization of residue-N, but lead to increased risk of higher mineral N loss at the same time. Therefore, we suggest that 75–25 is the best basal-topdressing ratio for N fertilizer application to promote the bioavailability of both urea and maize residue. Although N uptake from urea decreased when 25% urea-N was substituted with manure, there was no significant decrease observed for dry matter and total N uptake of wheat, suggesting that 25% was an acceptable percentage for organic substitution. One interesting finding is that the percentages of N in grain from fertilizer and residue were slightly higher than that in straw, which implies that N translocation inside the plant differs between N sources. It appears that exogenous N can be preferentially re-transported from straw to grain relative the indigenous soil N, and a similar finding was also reported by Gao et al. [31].

Soil N_min_ provides an immediate N source for crop demand, while soil MBN is a potential source of available N that can regulate N status in the soil and reduce N loss [43]. The averaged percentage of the soil total N pool derived from maize residue and urea was 4.2% and 5.5%, respectively, throughout the wheat growing stages (Table 4). Among these contributions, residue and urea contributed low levels (1.1% and 1.9%) to soil N_min_ pool and relatively high levels (5.9% and 11.7%) to the MBN pool. Lu et al. [9] reported that maize residue addition decreased the amounts of soil NH_4_^+^-^15^N and mineral-^15^N by 21.4% and 16.1% when compared to without maize residue, suggesting that combining N fertilizer with crop residue is an important means to reduce the superfluous accumulation of fertilizer-N to the soil N_min_ pool and it can subsequently reduce N loss. Determination of the PLFA and soil enzymes revealed that combined application of fertilizer and residue increased soil total microbial biomass and soil enzyme activities related to the C and N transformation (Fig 2; Table 5), which also explained this phenomenon. In some incubation experiments, there was rapid immobilization of soil and fertilizer-N during the first nine days after amendment with rice residue, which was gradually followed by N mineralization [44–45], similar to the results observed in the present study. The highest contribution to the soil MBN pool from the residue and urea occurred after dormancy of wheat, when the newly incorporated residue provided energy for microorganisms, resulting in a higher soil MBN content. However, the results observed by Shindo and Nishio [22] showed that nearly 10% of wheat residue-N was held in the MBN, regardless of the sampling time in an incubation experiment, implying that crop uptake may also induce a strong influence on N transformation in soil.

### Fate of ^15^N-labeled materials initially applied

The fate of fertilizer-N and residue-N was either absorbed by crops, remained in the soil, or was lost in various forms (N loss in our pot experiments only included NH_3_ volatilization and denitrification because non-leaking pots prevented leaching and runoff). We determined the recovery of ^15^N in grain, straw and roots of plants and the residual ^15^N in the soil, and the part not accounted for in the plant-soil system was presumably lost. We found that 35.9%, 28.1% and 36.0% of the ^15^N-labeled urea were absorbed by wheat, left in pots, and lost into the atmosphere, respectively during the first season (Fig 1B). As reported in a series of field microplot experiments, recovery, residual and loss rate of fertilizer-^15^N ranged 25–41%, 37–43% and 16–38% after the first-season crop harvest [34,46–47]. Poor synchronization between the availability of fertilizer N in soil and crop demand is responsible for low N use efficiency. Optimizing N regime has turned out to be an effective means of promoting the bioavailability of fertilizer-N, where fifty-fifty split application of urea promoted the utilization by decreasing the losses. Combined N losses through volatilization and leaching with glycine (39–53%) and ammonium sulfate (40–60%) fertilizers indicated both physicochemical and biological transformations of N by mineralization and nitrification [48]. However, gaseous N emissions were the only reason for high N losses in the present experiment. A comparable amount of N fertilizer (30%) was apparently lost from the soil-rice system, which was mainly attributed to ammonia volatilization in a pot experiment [49]. The combination of manure and urea decreased N recovery efficiency and decreased N loss compared to urea alone. Similar results were observed by Goloran et al. [50] and Chalk et al. [51], who found that recovery of applied ^15^N fertilizers by crops was higher without organic amendment compared to organic amendment (compost), and their ^15^N losses were also significantly reduced with organic amendment. These findings suggest that increased sorption or immobilization of soil mineral N following reduced N loss arises when organic residues are added.

Residue-N is actively involved in N transformation and cycling, and its release and recovery in plant-soil systems are controlled by the decomposition process. A review by Chalk et al. [51] showed that the ^15^N recovery from crop residues ranged from 12.4% to 19.1%, which was comparable to results of the present study. In the present study, 16.9%, 46.9% and 36.2% of ^15^N-labeled maize residue were absorbed by wheat, left in the soil, and lost into the atmosphere, respectively, across different N regime practices in the first season (Fig 1A). The great losses in residue-N in our study were attributed to a greater availability of carbon to the denitrifying population, which was demonstrated by the findings of soil enzyme activity and microbial biomass (Fig 2; Table 5). The recovery of crop residues appeared to be much lower relative to mineral fertilizer in the first season due to the relatively longer release process. N recovery of crop residues is heavily dependent on the residue quality, which is deemed to a primary controller of the decomposition rate, with faster decay associated with lower concentrations of chemically recalcitrant substrates such as lignin [35,52–53]. Crop residues higher in lignin with low N require additional N to increase decomposition, which soil microorganisms immobilize from the soil [54]. Therefore, mineral N management is another factor affecting the release of residue-N. We found the recovery of residue-N decreased with increased proportion of topdressing N, because the rapid decomposition occurs at the early stage of residues incorporated into the soil [35]. If more mineral N is added at the beginning to narrow the C/N ratios, the immobilization of residue-N will be mitigated and hence the bioavailability of residue-N will be promoted. When crop residues with wide C/N ratios were incorporated into soil, a large range from 4% to 20% of the applied residue N was reportedly recovered by crops in the first growing season [14,29,34,55–56]. However, opposed findings showed that air and soil temperatures are the driving force for residue decomposition, rather than N addition [57]. Definitely, temperature is one of the dominant factors affecting decomposition, especially in low-temperature and arid regions; but our finding confirmed the importance of N management on the decomposition of crop residues, and the key is to find an effective way for N application under residues retention.

### Residual effects of maize residue and urea

Although the majority of studies that determined fertilizer N recovery was limited to a single season, there are some in which the residual effects of fertilizer-^15^N have been measured and the recovery was reported being pretty low (< 10%) during the subsequent growing season(s) [29,31,58–59]. We found an average of 3.1% of the initially applied urea-^15^N was recovered in the second season, and this contributed 3.9% of the crop N, which was much lower than that in the first season (Fig 1B; Table 3). On one hand, the concentration of urea-^15^N in soil was diluted by additional unlabeled urea; on the other hand, residual urea-^15^N was immobilized into more stable organic matter. Thus, still 18.6% urea-^15^N was found left in the soil. Totally basal application of N fertilizer showed negative recovery of residual-^15^N, which should be an avoidable choice for N management practice.

Multi-year field experiments have illustrated that the cumulative N recovery of organic residues fitted well to a first-order kinetic model [60], and the curve appeared to reach a steady state after three years of cultivation [51]. In a 4-year microplot experiment, the cumulative recovery of the residue N increased gradually (18.2–20.9%), but most of the residue N was retained in the soil, notably in the 0–10 cm soil layer [14]. The residual availability of ^15^N-labeled residue and urea to subsequent summer maize indicated that the contribution of residue-N (averaged 3.0%) was slightly lower, but the recoveries of residue-N (averaged 4.1%) were higher than those of urea (Fig 1; Table 3). Despite the fact that the residual effects of crop residue-^15^N were not high, they were better than or equivalent to those of mineral fertilizer. Before the second season began, unlabeled wheat residue was incorporated into the soil. However, the sequential residue retention considerably enhanced the stabilization of the initially applied maize residue-^15^N in the soil and retarded its mineralization [14]. Therefore, there was still 29.3% residue-^15^N remaining in the soil after two seasons, which was much higher than that of urea-^15^N. Nevertheless, great losses from residual organic N occurred in the following season, requiring more effective management practices under the condition of continuously organic material inputs.

## Conclusions

To maximize the utilization of N fertilizer and organic residues, the first step is to identify their roles playing in crop growth. The findings from our pot experiments demonstrate that N contribution to crop uptake from maize residue, fertilizer, and the soil is approximately 1:3:6 with little change occurring across growth stages. As N fertilization regime has a strong influence on the availability of both mineral and organic N, deciding an appropriate basal-topdressing ratio for N application is important. However, there is a conflict between fertilizer-N and residue-N use efficiency: increasing proportions of topdressing N improved the bioavailability of fertilizer-N but decreased that of residue-N. A compromised basal-topdressing ratio of 75–25 for N spilt-application under maize residue retention may be suggested, whereas more detailed basal-topdressing ratios, such as 70–30 or 60–40, deserve to be further studied. Additionally, pot experiments have inherent limitations, thus, validating our findings under field conditions is necessary. Substituting 25% mineral N with manure is demonstrated as a valid means for decreasing chemical fertilizer input and loss, but negative responses are showed in the subsequent season. Therefore, how to promote the decomposition of large amounts of organic residues becomes urgent as increasing crop residues and organic fertilizers are returned to the fields worldwide.

## Supporting information

S1 Dataset The raw data of pot experiments. (XLSX)
